# Blocking LFA-1 Aggravates Cardiac Inflammation in Experimental Autoimmune Myocarditis

**DOI:** 10.3390/cells8101267

**Published:** 2019-10-17

**Authors:** Ludwig T. Weckbach, Andreas Uhl, Felicitas Boehm, Valentina Seitelberger, Bruno C. Huber, Gabriela Kania, Stefan Brunner, Ulrich Grabmaier

**Affiliations:** 1 Medizinische Klinik und Poliklinik I, Klinikum der Universität, LMU Munich, 81377 Munich, Germany; andreas.uhl@med.uni-muenchen.de (A.U.); felicitas.boehm@lrz.uni-muenchen.de (F.B.); valentina.seitelberger@med.uni-muenchen.de (V.S.); bruno.huber@med.uni-muenchen.de (B.C.H.); stefan.brunner@med.uni-muenchen.de (S.B.); ulrich.grabmaier@med.uni-muenchen.de (U.G.); 2 Walter Brendel Centre of Experimental Medicine, University Hospital, LMU Munich, 82152 Planegg-Martinsried, Germany; 3 Institute of Cardiovascular Physiology and Pathophysiology, Biomedical Center, LMU Munich, 82152 Planegg-Martinsried, Germany; 4 German Center for Cardiovascular Research, Partner Site Munich Heart Alliance, 80802 Munich, Germany; 5 Center of Experimental Rheumatology, Department of Rheumatology, University Hospital Zurich, 8952 Schlieren, Switzerland; gabriela.kania@uzh.ch

**Keywords:** myocarditis, inflammation, leukocytes

## Abstract

The lymphocyte function-associated antigen 1 (LFA-1) is a member of the beta2-integrin family and plays a pivotal role for T cell activation and leukocyte trafficking under inflammatory conditions. Blocking LFA-1 has reduced or aggravated inflammation depending on the inflammation model. To investigate the effect of LFA-1 in myocarditis, mice with experimental autoimmune myocarditis (EAM) were treated with a function blocking anti-LFA-1 antibody from day 1 of disease until day 21, the peak of inflammation. Cardiac inflammation was evaluated by measuring infiltration of leukocytes into the inflamed cardiac tissue using histology and flow cytometry and was assessed by analysis of the heart weight/body weight ratio. LFA-1 antibody treatment severely enhanced leukocyte infiltration, in particular infiltration of CD11b+ monocytes, F4/80+ macrophages, CD4+ T cells, Ly6G+ neutrophils, and CD133+ progenitor cells at peak of inflammation which was accompanied by an increased heart weight/body weight ratio. Thus, blocking LFA-1 starting at the time of immunization severely aggravated acute cardiac inflammation in the EAM model.

## 1. Introduction

Myocarditis is a major cause of heart failure in young adults. Infections with cardiotropic viruses represent the most common etiology of myocarditis in the Western World. Release of cardiac self-antigens can subsequently lead to breakdown of heart-specific tolerance tissue and can evolve into autoimmune-mediated inflammation sustaining the disease upon eradication of the virus. Sustained cardiac inflammation may eventually lead to cardiac remodeling and end-stage heart failure with dilation of the ventricles and deteriorating contractility of the cardiac muscle, a condition called inflammatory dilated cardiomyopathy (DCMi) [1]. We recently showed that neutrophils play a critical role for cardiac inflammation in the experimental autoimmune myocarditis (EAM) mouse model which resembles the immunological and histopathological features of post-viral heart disease [2,3]. In this model, cardiac inflammation was induced by administration of a cardiac peptide together with complete Freund’s adjuvant which triggered an autoimmune response with a peak of leukocyte infiltration at day 21 after immunization. We demonstrated that targeting neutrophil extracellular traps (NETs), a process by which neutrophils expel their nuclear DNA together with antibacterial proteins, thereby maintaining tissue inflammation, can substantially reduce cardiac inflammation in the EAM mouse model [2,4]. In order to undergo NET formation in the inflamed cardiac tissue, neutrophils must be recruited from the blood stream into the tissue by crossing the endothelial barrier of the cardiac vasculature. The neutrophil recruitment cascade consists of consecutive events including capturing of free-flowing neutrophils, rolling, adhesion, adhesion strengthening, intraluminal crawling, and transmigration [5]. The lymphocyte function-associated antigen 1 (LFA-1, CD11a/CD18) and the macrophage 1-antigen (Mac-1, CD11b/CD18), adhesion molecules of the beta2-integrin family, are of fundamental importance for neutrophil adhesion and subsequent recruitment into the inflamed tissue [6]. LFA-1 also acts as an important adhesion molecule for other leukocyte subsets during the recruitment process, e.g., for T cells. Beyond its role for leukocyte recruitment, the interaction of LFA-1 on T cells with its ligand (the intercellular molecule-1 (ICAM-1)) on antigen-presenting cells like dendritic cells can provide a co-stimulatory signal for T cell activation. It has been shown that LFA-1 signaling influences differentiation of T cells into specific effector subsets. Engagement of LFA-1 on T cells can activate the Notch pathway promoting Th1 differentiation and suppressing generation of Th17 cells and regulatory T cells [7]. LFA-1 has been investigated as target in different autoimmune diseases. In psoriasis, an antibody targeting LFA-1 resulted in significant improvement of plaque psoriasis in patients with moderate to severe disease [8]. In contrast, in the experimental autoimmune encephalomyelitis (EAE) model, infiltration of leukocytes into the spinal cord and the brain was substantially enhanced in LFA-1^-/-^ mice which was accompanied by increased disease severity suggesting that LFA-1 was protective in this model [9]. The role of LFA-1 in myocarditis is unclear. In this study, we set out to investigate the role of LFA-1 in myocarditis using the EAM model.

## 2. Materials and Methods

### 2.1. Mice

Male wild-type mice (8 weeks) on a BALB/c background were obtained from Charles River (Sulzfeld, Germany). Animals were fed a standard chow diet ad libitum with free access to water. All animal experiments were approved by the Regierung von Oberbayern (55.2-1-54-2532-48-2014), Germany.

### 2.2. EAM Model

To induce EAM, a purified synthetic peptide of the cardiac myosin heavy chain alpha (Ac-RSLKLMATLFSTYASADR, Caslo, Kongens Lyngby, Denmark) emulsified in complete Freund’s adjuvant (CFA, Sigma-Aldrich, St. Louis, MO) was applied subcutaneously (200 µg of cardiac peptide per mouse) at day 1 and day 7. Equal volumes of CFA + PBS were administered for sham controls. In order to block LFA-1, we used a chimeric rat-mouse IgG1 anti-mouse CD11a monoclonal antibody (muM17, Genentech, San Francisco, CA) which was previously described [10]. The antibody was applied subcutaneously (5 µg/g body weight) starting from day 1 once a week until day 21. Control animals were treated with a matching IgG1 isotype antibody (Genentech, San Francisco, CA) or PBS. Sham-immunized controls were treated with PBS. On day 21, mice were sacrificed and hearts were subsequently removed for analysis.

### 2.3. Histology and Heart Weight/Body Weight Ratio

To evaluate the cardiac tissue histologically, mouse hearts were rinsed with PBS and fixated with PFA 4% (Roth, Karlsruhe, Germany). Analysis of the heart weight/body weight ratio was conducted using a microbalance (CP64-0CE, Sartorius, Göttingen, Germany) after carefully removing the pericardium, connective tissue, and vascular remains. Thereafter, hearts were dehydrated in a graded series of ethanol concentrations and subsequently embedded in paraffin (Sigma-Aldrich, St. Louis, MO, USA). To evaluate infiltration of leukocytes, sections were stained with hematoxylin (Roth) and eosin (Roth, H&E, day 21). The established EAM score (0: no inflammatory infiltrates; 1: small foci of <100 inflammatory cells between myocytes; 2: larger foci of >100 inflammatory cells; 3: >10% of a cross section shows infiltration of inflammatory cells; 4: >30% of a cross section shows infiltration of inflammatory cells) was used to evaluate leukocyte infiltration semi-quantitatively as previously described [2]. Analysis was performed in a blinded manner.

### 2.4. Flow Cytometry

To study infiltration of different leukocyte subsets into the cardiac tissue, flow cytometry was performed at day 21. The hearts were removed, perfused with PBS, subsequently cut in small pieces, and incubated with Liberase (Roche, Basel, Switzerland) for 45 min at 37 °C. Next, the suspension was mixed gently, filtered through a 40 µm cell strainer, and subsequently suspended in PBS. Cells were stained with a APC-conjugated rat anti-mouse CD11b antibody (clone M1-70, BD, Franklin Lakes, NJ), PE-conjugated rat anti-mouse Ly6G antibody (clone 1A8, Biolegend, San Diego, CA, USA), PerCP-conjugated rat anti-mouse CD45 antibody (clone 30F-11, BD), PB-conjugated rat anti-mouse CD4 antibody (clone RM4-5, BD), an APC rat anti-mouse F4/80 antibody (clone BM8, Biolegend) and FITC-conjugated rat anti-mouse CD133 antibody (clone EMK08, ThermoFisher, Waltham, MA, USA). Experiments were performed using a Gallios flow cytometer (Beckman Coulter, Krefeld, Germany). FlowJo software (TreeStar, Ashland, OR) was used to analyze data.

### 2.5. Statistical Analysis

Data shown represent the mean ± SEM. A Kolmogorov-Smirnoff test was conducted to test for normal distribution. As data were not normally distributed, a Kruskal Wallis test with pairwise comparison and Dunn-Bonferroni correction for multiple testing was applied for all statistical tests. An alpha level of 5% was considered as statistically significant. All data was analyzed using SPSS version 26.

## 3. Results

**Figure 1:**
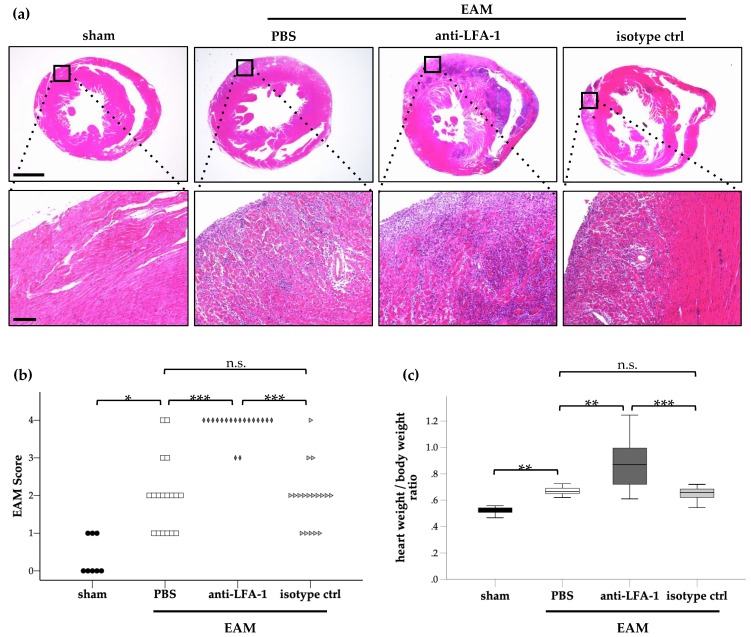
Blocking lymphocyte function-associated antigen 1 (LFA-1) aggravated cardiac inflammation in experimental autoimmune myocarditis (EAM): (**a**) Representative cross sections of heart tissue of sham-treated control mice, vehicle-treated EAM mice (PBS), anti-LFA-1-treated EAM mice as well as isotype control-treated mice on day 21. (**b**) EAM score (**c**) as well as evaluation of the heart weight/body weight ratio at day 21 after induction of EAM (EAM) or sham immunization (sham). EAM mice were treated with an anti-LFA-1 antibody (anti-LFA-1), a matching isotype control (isotype ctrl), or PBS as indicated, with *n* = 8 for sham group; *n* = 18 for EAM groups; * *p* < 0.05; ** *p* < 0.01; *** *p* < 0.001; n.s., not significant. Kruskal Wallis test followed by Dunn-Bonferroni post hoc test. Data are presented as (**b**) individual data points or (**c**) median with interquartile range, whiskers indicate 95% confidence interval.

To investigate the role of LFA-1 for cardiac inflammation in myocarditis, we evaluated leukocyte infiltration and the heart weight/body weight ratio in the EAM model after blocking LFA-1. We targeted LFA-1 using a chimeric rat-mouse anti-mouse antibody from day 1 until day 21 during the course of EAM. Leukocyte infiltration into the inflamed cardiac tissue, which was analyzed histologically using a semi-quantitative score (referred to as EAM score), was significantly increased at day 21 in immunized mice without antibody treatment (PBS) or with isotype control antibody treatment compared to sham-immunized mice, as expected. Strikingly, leukocyte infiltration was substantially enhanced after blocking LFA-1 compared to mice treated with PBS or the matching isotype control antibody suggesting that LFA-1 suppressed leukocyte infiltration into the inflamed cardiac tissue in EAM (Figure 1a,b). The heart weight/body weight ratio was altered accordingly (Figure 1c).

**Figure 2:**
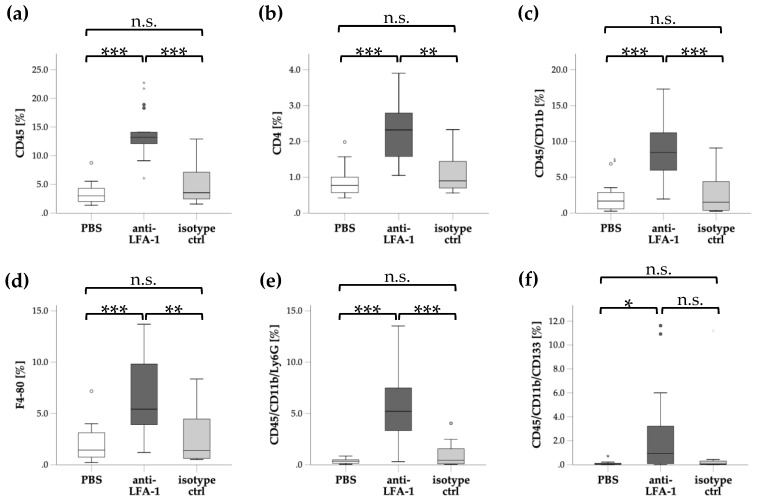
Infiltration of different leukocyte subsets is enhanced by blocking LFA-1: Flow cytometric analysis of leukocyte subpopulations in the inflamed cardiac tissue on day 21 after induction of EAM. Diagrams display the percentage of leukocytes (**a**, CD45) and leukocyte subpopulations (**b**, CD4; **c**, CD45/CD11b; **d**, F4-80; **e**, CD45/CD11b/Ly6G; **f**, CD45/CD11b/CD133) of all cells after blocking LFA-1 (anti-LFA-1) compared with the matching isotype control antibody (isotype ctrl) or PBS. *n* = 18 for PBS, *n* = 17 for anti-LFA-1 and isotype ctrl. * *p* < 0.05; ** *p* < 0.01; *** *p* < 0.001; n.s., not significant. Kruskal Wallis test followed by Dunn-Bonferroni post hoc test. Data are presented as median with interquartile range, whiskers indicate 95% confidence interval.

Next, we determined whether infiltration of specific leukocyte subsets into the cardiac tissue in EAM would be affected by the blockade of LFA-1 in EAM. For this purpose, we administered the LFA-1 blocking antibody or the matching isotype or PBS for control from day 1 until day 21 and subsequently determined the percentage of infiltrated leukocyte subsets of all cells using flow cytometry. The percentage of infiltrated leukocytes determined by CD45+ cells was substantially enhanced by blockade of LFA-1 compared to PBS or the isotype control antibody (Figure 2a). To evaluate the specific leukocyte subsets affected by targeting LFA-1, we also stained for CD4, CD11b, F4-80, Ly6G, and CD133. Blockade of LFA-1 significantly increased the infiltration of all CD4+ T cells (Figure 2b). Moreover, infiltration of CD45/CD11b cells (mainly consisting of monocytes and neutrophils) was significantly elevated in LFA-1 antibody-treated mice compared to control mice (Figure 2c). Accordingly, an increased percentage of infiltrated F4-80+ cells resembling monocytes/macrophages was observed after blocking LFA-1 compared to control conditions (Figure 2d). Moreover, LFA-1 antibody treatment led to a higher number of neutrophils in the inflamed cardiac tissue (Figure 2e). Finally, the fraction of CD133+ progenitor cells, which represent the cellular source of TGF-β mediated fibrosis, was increased after LFA-1 blockade compared to PBS control albeit not reaching a statistical significant difference compared to isotype control (Figure 2f). These findings imply that blocking LFA-1 substantially promoted leukocyte infiltration into the inflamed cardiac tissue affecting CD4+ T cells, monocytes/macrophages, neutrophils, and possibly profibrotic CD133+ progenitor cells.

## 4. Discussion

Blocking LFA-1 or the absence of LFA-1 in different autoimmune diseases revealed a protective or detrimental role of this adhesion receptor depending on the autoimmune disease [8,9]. Moreover, the time-point of targeting LFA-1 may also have an enormous impact on the course of the particular disease. Our findings showed that biological blockade of LFA-1 from day 1 until day 21 substantially enhanced leukocyte infiltration into the inflamed cardiac tissue in the EAM model compared to immunized mice treated with an isotype antibody or PBS. Infiltration of inflammatory cells was accompanied by a dramatic increase of the heart weight/body weight ratio which is a very robust marker for cardiac inflammation indicating that blocking LFA-1 significantly promoted acute myocarditis in this model. Interestingly, all immunized mice treated with the anti-LFA-1 antibody showed the full phenotype of myocarditis (EAM score ≥ 3) whereas a significant percentage of mice generally develop only mild disease (EAM score ≤ 1). Analysis of different leukocyte subpopulations revealed that infiltration of CD4+ T cells, monocytes/macrophages, and neutrophils was enhanced after blocking LFA-1 compared to control conditions suggesting that all investigated leukocyte subsets were affected. In addition, we observed a numerical but not statistical significant increase of CD133+ progenitor cells after targeting LFA-1 which have been shown to promote fibrosis by expression and release of TGF-β [11]. These findings may point to a profibrotic phenotype after blocking LFA-1 in the EAM model.

The underlying mechanism of our preliminary findings remains unclear. As described above, LFA-1 serves as adhesion receptor in particular for neutrophils and T cells. In a peritonitis mouse model, blocking LFA-1 significantly reduced neutrophil recruitment into the peritoneal cavity [12]. The contribution of LFA-1 for neutrophil adhesion and subsequent recruitment depend on the tissue and the predominating inflammatory stimuli in the particular inflammatory setting [13]. The importance of LFA-1 for T cell adhesion and extravasation is also tissue-dependent as T cells also use the very late antigen-4 (VLA-4) as adhesion receptor [14]. Furthermore, the relevance of VLA-4 or LFA-1 may also depend on the T cell subset in specific inflammatory settings [15]. However, as we did not observe reduced but substantially enhanced CD4+ T cell infiltration, we did not assume reduced recruitment of a specific T cell subset in our model.

In line with our study, aggravated leukocyte infiltration was observed in LFA-1-deficient mice in the EAE model which was caused by an impairment of the generation of regulatory T cells and subsequent expansion in the absence of LFA-1 [9]. Enhanced CD4+ T cell infiltration into the spinal cord was also observed by targeting LFA-1 in the EAE model [15]. Whether blocking of LFA-1 also impacted expansion of regulatory T cells in the EAM model requires further investigation.

As described above, LFA-1 engagement can promote Th1 cell and suppress Th17 cell differentiation. Interferon γ-producing Th1 cells represent the predominant T cell subset in autoimmune myocarditis [16]. However, depletion of IL-17 reduced severity but did not prevent EAM, suggesting that both subsets contribute to the inflammatory milieu in the EAM model [17]. Blocking LFA-1 could potentially impact the fate of T cells towards Th17 effector cells thereby changing the inflammatory setting. However, the mechanism of enhanced cardiac inflammation in myocarditis after blocking LFA-1 still needs to be determined.

In summary, targeting LFA-1 substantially enhanced infiltration of neutrophils, monocytes/macrophages, CD4+ T cells and potentially CD133+ progenitor cells and thereby promoted cardiac inflammation in the EAM model. These findings suggest that engagement of LFA-1 may prevent excessive inflammation in myocarditis.
